# The Handsome Cross Grasshopper *Oedaleus decorus* (Germar, 1825) (Orthoptera: Acrididae) as a Neglected Pest in the South-Eastern Part of West Siberian Plain

**DOI:** 10.3390/insects13010049

**Published:** 2022-01-01

**Authors:** Kristina V. Popova, Natalya S. Baturina, Vladimir V. Molodtsov, Oxana V. Yefremova, Vasily D. Zharkov, Michael G. Sergeev

**Affiliations:** 1 Department of General Biology and Ecology, Novosibirsk State University, 2 Pirogova Street, 630090 Novosibirsk, Russia; kristin_belle@mail.ru (K.V.P.); natalya.s.baturina@gmail.com (N.S.B.); vv@fen.nsu.ru (V.V.M.); oxana@fen.nsu.ru (O.V.Y.); arthropodae01@gmail.com (V.D.Z.); 2 Laboratory of Invertebrate Ecology, Institute of Systematics and Ecology of Animals, Siberian Branch, Russian Academy of Sciences, 11 Frunze Street, 630091 Novosibirsk, Russia

**Keywords:** distribution, range, dispersal, South Siberia, dynamics, population, plant protection, outbreak, Italian locust

## Abstract

**Simple Summary:**

The handsome cross grasshopper is one of the most abundant and widely distributed grasshopper species over the steppe and semi-desert territories of Eurasia. In many areas, especially in east Mongolia and north-east China, it is a very common and dangerous pest on agriculture fields and pasturelands. However, the species was relatively rare in the steppes of west Siberia until the 1960s, but by the end of the last century, the geographic and ecological distribution of the handsome cross grasshopper was changed significantly. Nowadays, it often occurs across all steppes of the south-eastern part of West Siberian Plain, its abundance is high, and its populations are found in the forest-steppes and also on the eastern side of the Ob River. The authors tried to use ecologo-geographic modelling to estimate how species distribution may change in the near future.

**Abstract:**

*Oedaleus decorus* is a widely distributed acridid over the Eurasian semi-arid territories, from the Atlantic coast to the Pacific coast. In many semi-arid territories, *O. decorus* was and is the most important pest, but in the south-eastern part of West Siberian Plain, it was not considered a pest until the 1960s. We compared two sets of data on the acridid distribution in the region: before 1960 and from 1961 until 2021. Until the 1960s, the species occurred mainly in the southern steppes. Since the 1960s, its distribution changed significantly. Nowadays, it occupies almost all local steppes and the southern part of the forest-steppes and can be also found on the eastern side of the Ob River. These shifts may be explained by both climatic changes and changes in human activities. During upsurges the densities of *O. decorus* were often more than one to two adults per square meter. It is often abundant in the same habitats and in the same periods as the Italian locust (*Calliptamus italicus*)—one of the most important acridid pests. This means during joint outbreaks these two species can simultaneously damage almost all spectrum of plants.

## 1. Introduction

The south-eastern part of West Siberian Plan lies in the central section of the range of *Oedaleus decorus* (Germar, 1825) or the handsome cross grasshopper [1,2]. This acridid species is widely distributed over the Eurasian semi-arid areas (mainly across forest-steppes, steppes, semi-deserts, Mediterranean and mountain dry grasslands), from the Atlantic coast to the Pacific one [3,4,5,6,7,8]. It also occurs in North Africa, on the Canary Islands, and on Madeira [3]. *O. decorus* commonly prefers different dry grasslands, from dry meadows to some types of the deserts with grasses [9,10,11,12]. The species is usually univoltine with overwintering eggs. It prefers to feed on different grasses and commonly avoids forbs [12,13,14,15,16,17]. During outbreaks, it can seriously damage cereals, pastures, and hayfields [13,18,19], and it may also harm cotton, alfalfa, different legumes, sugar beet, vegetables, vine, and some fruit trees [13]. In many semi-arid territories, *O. decorus* was and is the important pest [18,19,20,21], especially in the eastern part of its range [22,23,24]. However, in the south-eastern part of West Siberian Plain, one of the main agricultural regions of Russia and Kazakhstan, according Berezhkov [25], until the 1960s this species occurred mainly in the dry (southern) steppes, its abundance was low, and it was not considered as a pest (cf. [19]). Furthermore, in many cases, plant protection services did not and do not still separate grasshoppers’ species when they collect data concerning acridid abundance. During the twentieth century, ecosystems of this region were significantly changed by human activity. For instance, in 1954–1956 about 4.4 million ha were ploughed in the Altaj and Novosibirsk Regions during the so-called Virgin Land campaign [26]. Later, in the end of the 20th century, the general area of arable fields significantly decreased from about 11 million to 10.2 million ha (for two regions) [27]. In the second half of the last century, such ecosystems’ transformations and, perhaps, global climatic variations as well resulted in species distribution changes [1,2,28]. The aims of this publication are to show how the distribution of *O. decorus* over West Siberian Plain has shifted, to estimate what climatic factors are essential for the species, how its distribution can change in the coming years, and to discuss some forecasts of its dispersal until 2040.

## 2. Materials and Methods

### 2.1. Study Territory

Original data were collected from 1979 until 2021 in the south-eastern part of West Siberian Plain. We also used some additional data collected in the adjacent north-eastern and eastern parts of the Kazakh Uplands (Saryarqa) and in the steppes on the western side of the Irtysh River in 1980. The south-eastern part of West Siberian Plain borders the Irtysh River to the west and south-west and the mountains of south Siberia to the east and south-east. Its northern boundary is approximately defined by the southern border of the taiga life zone (about 56° N). Originally this area was characterized by grasslands and forests. Later it was mainly converted to agricultural lands (fields and pastures). The forest-steppe life zone occupies mainly the area from 54° N to 55.5° N between the Irtysh and Ob Rivers and also on the eastern side of the Ob River. The steppes are between 50.5° N to 54° N, but in the western and central parts of the region. There are some flood-plains with meadows and forest patches, sandy plots, solonchaks (with saline soils), and swamps as well. Average temperatures are relatively low (mean temperatures of the warmest month are between 17 to 22 °C, the same for the coldest month—from −17 to −20 °C), and annual precipitation amounts range between 280 to 520 mm [29]. 

### 2.2. Field Studies

Grasshoppers’ distribution patterns were characterized by quantitative and qualitative samples collected in natural and transformed ecosystems, usually in July and August when adults were dominated [4,28]. Samples captured during a fixed period of time were done in every habitat studied [4,30,31]. Using this method, insects were collected with a standard net (40 cm diameter) over a period of 10–30 min. Results for each habitat were transformed to an hour. As a rule, hand collecting and sweep netting were used to get additional information as well. In many cases, especially during locust outbreaks, we also counted acridid densities on arbitrarily placed plots 0.25 × 0.25 m^2^ (in some cases—0.5 × 0.5 m^2^), mainly in the zonal habitats [31,32]. We used the GLONASS/GPS navigators to determine geographical coordinates of localities.

Several fixed sites were selected to study long-term dynamics of the species populations [32,33]. All plots were covered by more or less typical steppe zonal vegetation; however, in some cases, vegetation cover was damaged by moderate grazing or haymaking. As a rule, the acridid abundance was counted in the first half and middle of July, while adults prevailed. In this study, we analyzed data for 2000–2008, 2015, and 2018 when all three sites were explored (except of Yarovoe in 2018).

1. SE Aleksandrovskij settlement (Novosibirskaya Oblast (Novosibirsk Region), 53.67° N 78.25° E, northern steppe, in 2003 the local control model plot was moved about 100 m southwards, because the main part of the area was ploughed).
2. SW Yarovoe town (Altai Krai (Altaj Region), 52.85° N 78.57° E, dry steppe (actually very old crested wheatgrass field)).
3. E Ust-Volchikha settlement (Altai Krai (Altaj Region), 51.93° N 80.28° E, dry steppe).

In 2004, several series of adults of *O. decorus* were specially collected in some localities of the Kulunda steppe as well to compare some phenotypic variations of its local populations. 

### 2.3. Data Analysis

Besides our original data, we also analyzed some old data, especially collected by the expeditions of Novosibirsk State University (1961–1980), and, in this case, checked some previous species identifications. These samples were collected by the methods described in Section 2.2. For localities explored before 2000, we used Google Earth Pro (©Google, 2020) to get geographical coordinates of localities. The main part of studied specimens is in the collections of Novosibirsk State University and the Institute of Systematics and Ecology of Animals (Novosibirsk). We also used data from different publications for the end of the 19th century [34] and the first half of the 20th century [25,35,36,37,38,39,40,41] and data for some specimens from the collections of Zoological Institute, Russian Academy of Sciences (Saint Petersburg, Russia), Novosibirsk State University, and Berezhkov’s collections at Tomsk State University (Tomsk, Russia). The geographic coordinates for almost all old localities of grasshoppers (151) were determined (Table S1). These data were compared with dataset for 1961–2021 (194 localities for all acridid insects) (Table S2). 

Maps of species distribution were produced on the basis of geographic coordinates with MapInfo 15.2.4 (© Pitney Bowes Software Inc., Lanham, MD, USA; now—© Precisely, Burlington, MA, USA). A Lambert conformal conic projection (ETRS 89) was used as the basic map.

We used Maxent 3.4.4 software [42,43,44,45] to model the species distribution over the south-eastern part of West Siberian Plain for two sets of data, until 1960 and from 1961 until 2021. We selected this software, because it is highly standardized [43] and has a relatively friendly interface. 

For ecomodelling, we exploited resources of WorldClim 2 [46,47] such as “Historical climate data” (19 standard annually averaged bioclimatic variables and 12 monthly averaged variables for solar radiation for 1970–2000 at the 30 arcsecond spatial resolution) and “Future climate data” (19 standard averaged bioclimatic variables for 2021–2040 downscaled from two global climate models [47], namely CNRM-ESM2-1 (Centre National de Recherches Meteorologiques and Centre Europeen de Recherche et de Formation Avancee en Calcul Scientifique, France) [48,49] and MIROC6 (Japan Agency for Marine-Earth Science and Technology, Atmosphere and Ocean Research Institute, The University of Tokyo, National Institute for Environmental Studies, and RIKEN Center for Computational Science, Japan) [50,51], at the 2.5 arcminute spatial resolution and for the 3–7.0 Shared Socioeconomic Pathway based on high greenhouse gas emissions [52]). 

Both the approach to ecomodelling and the data on climatic variables have some limitations. The MaxEnt models are based only on presence data and depend on the number of occurrences, selected parameters of modelling, and selected sets of variables [42,43,44]. These limitations can be especially important for an analysis of small samples [53]. Besides, the WorldClim dataset includes spatially interpolated climatic data, and their reliability depends partly on densities of weather stations (relatively low for south Siberia) [46]. This is why results of our ecomodelling should be qualified as the preliminary ones. In any case, we tried to use the full sets of applicable bioclimatic variables to compare results for the same territory, but for different periods, to estimate accuracy of our models by using the AUC (the area under the receiver operating characteristic curve) values for training and test (25%) data and producing sets of 20 replicates with cross-validation, and to estimate significance of climatic variables by their predictive contributions and Jackknife tests. We generated the models with the following parameters: features—auto, output format—cloglog [44], regularization multiplier = 1. 

The Spearman rank-order correlation coefficient was used to compare long-term dynamics patterns of local populations of *O. decorus* and the Italian locust (*Calliptamus italicus* (Linnaeus)). We also analyzed several series of the handsome cross grasshopper adults, including at least five specimens and estimated frequencies of brown and green morphs relative to the species abundance, because in the populations of the so-called Mongolian locust (*Oedaleus decorus asiaticus* Bey-Bienko), the brown morphs are mainly associated with high levels of species abundance and the green ones are common at low density [54]. We used a multivariate regression analysis to compare several rows of data as well. These parts of the analysis were mainly conducted using PAST 4.02 [55].

## 3. Results

### 3.1. Taxonomical Notes

In the first half of the 20th century, some specimens of the genus *Oedaleus* Fieber from the West Siberian Plain were misidentified as *O. infernalis* Saussure [25]. We checked several such specimens from Berezhkov’s collections (Tomsk State University, Tomsk, Russia). All of them proved to be *O. decorus*. To distinguish similar specimens and the true individuals of *O. infernalis* from East Asia, Bey-Bienko [56] described the new species, namely, *O. asiaticus* Bey-Bienko, from South Siberia. Later Ritchie [3] revised the genus and showed that *O. asiaticus* is conspecific with *O. decorus*. He noted very significant variability of individuals of *O. decorus* from different parts of its range. Some orthopterists [4,21,57,58] follow this proposal, and our data support this point of view [59]. However, some others [60] do not. Molecular data do not allow us to solve this problem up to date. Fries et al. [61] showed that *O. decorus* and *O. asiaticus* are very close forms, but regarded them as separate species. Kindler et al. [62] discussed that this species complex includes three distinct, but closely related species or subspecies. Later, Schmid et al. [63] also described evident similarity between *O. decorus decorus* from Europe and *O. decorus asiaticus*. That is why we prefer to follow the most comprehensive revision of the genus [3,59].

### 3.2. Shifts in Species Distribution

**Figure 1:**
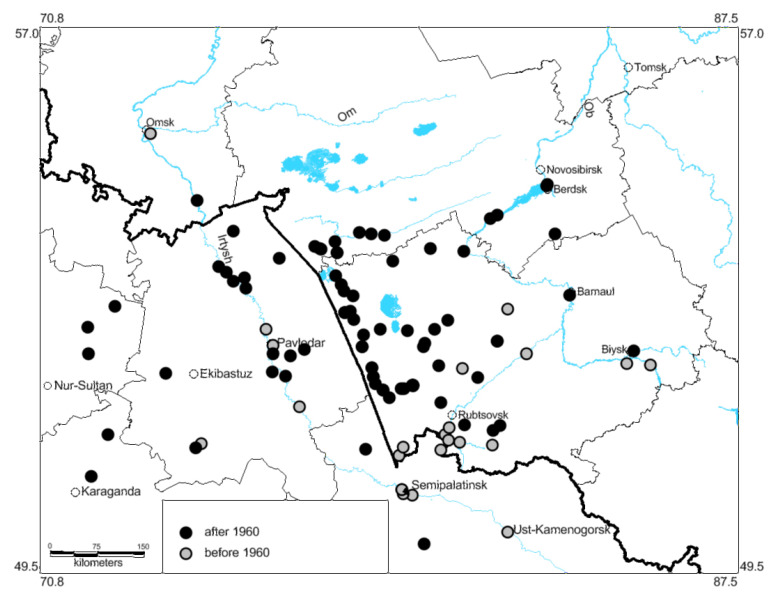
Distribution of *Oedaleus decorus* over the south-eastern part of the West Siberian Plain until 1960 and after 1961.

**Figure 2:**
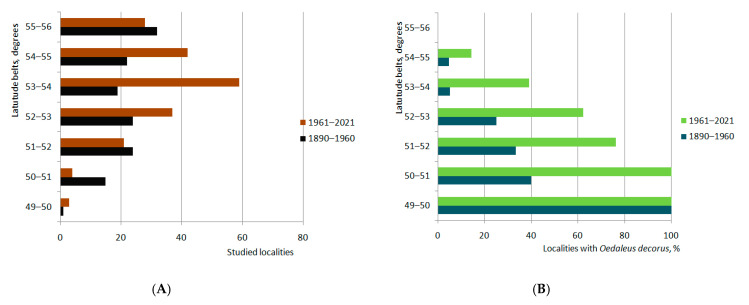
Latitudinal distribution of all studied localities (**A**) and the localities with *Oedaleus decorus* (**B**) over the south-eastern part of West Siberian Plain until 1960 and after 1961 (Tables S1 and S2).

**Figure 3:**
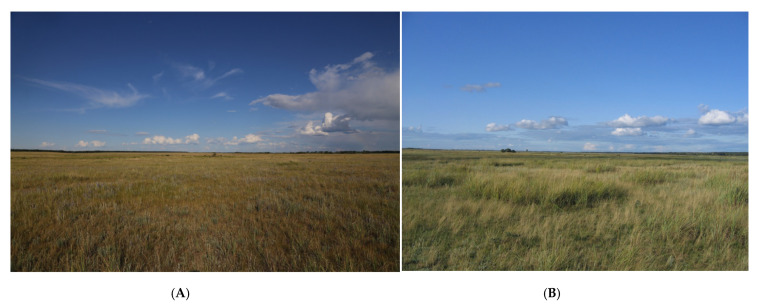
Typical habitats of *Oedaleus decorus* in the Kulunda steppe: (**A**)—typical steppe (near the Kuchuck River, Altai Krai (Altaj Region), 52.41° N 80.03° E); (**B**)—dry steppe (near Ust-Volchikha settlement, Altai Krai (Altaj Region), 51.93° N 80.28° E).

Until the middle of the 20th century, *O. decorus* was mainly distributed in the southern parts of the region (Figure 1 and Figure 2) and was commonly associated with the dry steppes (Figure 3B). Its northernmost localities were found near the 53th parallel north in the so-called Kulunda steppe between the Irtysh and Ob Rivers [25]. The only known exception was the locality in the vicinities of Omsk (about 55° N—the forest-steppes of the Irtysh River basin) [37,40,41]. Bey-Bienko [41] found this species near Omsk on the dry southern slope on the eastern side of the Irtysh River and noted that it was very rare.

**Figure 4:**
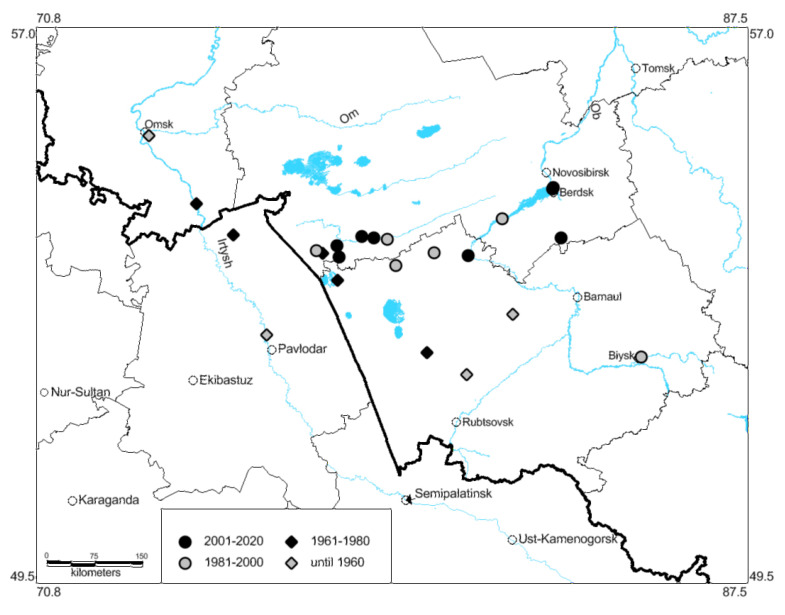
Changes of the known northernmost and north-easternmost localities of *Oedaleus decorus* in the south-eastern part of the West Siberian Plain from the beginning of the 20th century.

Our data show that in the 1970s, the species occurred already over the whole Kulunda steppe (up to 54° N) (Figure 1 and Figure 4). In the 1990s, *O. decorus* crossed the 54th parallel, and its populations were observed in the southern parts of the forest-steppe. In 1999, its specimen was found in the southern part of Novosibirsk, in Novosibirsk Scientific Center of Russian Academy of Sciences (so-called Akademgordok) [64], on the eastern side of the Ob River. This locality became the north-easternmost one on the West Siberian Plain. Later, several colonies of this species were observed in the forest-steppes on the eastern side of the Ob River. They were commonly associated with dry transformed habitats, e.g., with overgrazed meadow steppes, lawns, and roadsides. 

The multivariate regression analysis of species occurrences relative to latitudinal bands (Figure 2) shows very significant correlations between these parameters for each period: until 1960 (r = –0.888, *p* = 0.008) and from 1961 until 2021 (r = –0.987, *p* = 0.00004) and explicit difference between two periods (overall statistics: R^2^ = 0.895, F = 522.6, MSE = 175.8, Wilks’ lambda = 0.0038). 

Hence, *O. decorus* has become one of the most widely distributed grasshopper over the south-eastern parts of West Siberian Plain and occupies all steppe areas, the southern parts of the forest-steppes between the Irtysh and Ob Rivers, and the forest-steppes on the eastern side of the Ob River where it colonized some transformed habitats [1,2].

### 3.3. Ecological Models of the Species Distribution

**Figure 5:**
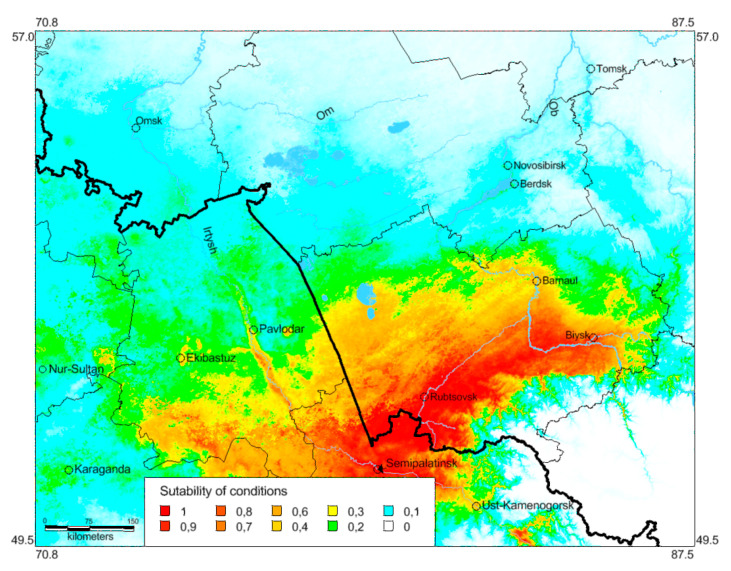
Predicted probabilities of suitable conditions for *Oedaleus decorus* in the south-eastern part of the West Siberian Plain (distribution data before 1961 and bioclimatic variables for 1970–2000; point-wise mean for 20 replicates).

The analysis of the predicted distribution of *O. decorus* based on the occurrence data until 1960 relative to climatic data for 1970–2000 (Figure 5) allows us to compare such backward forecasts with the actual distribution of species in the end of the 20th century. This analysis shows that (1) its northernmost population (for this period) near Omsk (Figure 1, Figure 2 and Figure 4) looks like the very local and limited one [41] (perhaps temporal), (2) conditions for this species may be suitable not only in the southern part of the Kulunda steppe, but in its central and eastern territories as well and also in some areas on the eastern side of the Ob River. Perhaps, the old mention of *O. infernalis* for Meret settlement (now in Novosibirsk Region near the boundary of Altaj Region, on the eastern side of the Ob River, 53.57° N 82.40° E) [65] really belongs to *O. decorus*. If this is right, the first colony (or colonies) of the species occurred on the eastern side of the Ob River more than a century ago. 

This model shows that in the end of the 20th century, global warming could result in the species distribution shifts northward and north-eastward. However, the actual distribution pattern (Figure 1) indicates that *O. decorus* occupied all local steppes of the Altaj, Novosibirsk, Omsk, and Pavlodar Regions and penetrated into the southern parts of the forest-steppes in the Novosibirsk Region. It also became widely distributed across the forest-steppes on the eastern side of the Ob River. Such deviations can be partially explained by species dispersal through transformed habitats, e.g., roadsides, abandoned fields, overgrazed meadow steppes, dry lawns. 

**Figure 6:**
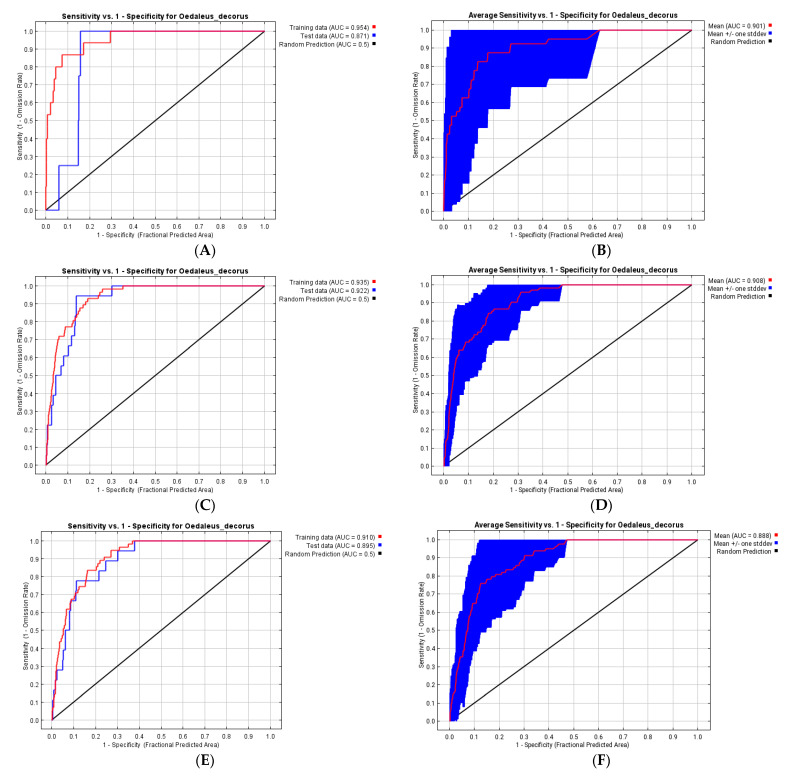

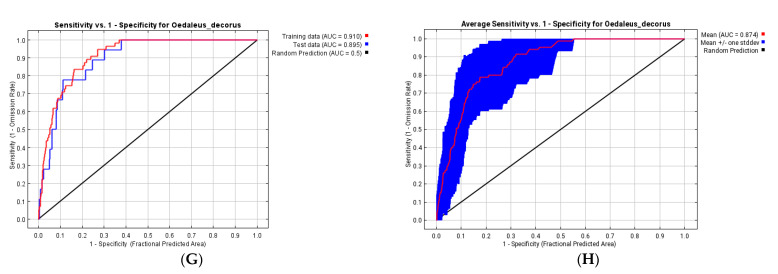
Reliability tests of the *Oedaleus decorus* distribution models for the south-eastern part of the West Siberian Plain: (**A**,**B**)—distribution data before 1961 and bioclimatic variables for 1970–2000; (**C**,**D**)—distribution data from 1961 until 2021 and bioclimatic variables for 1970–2000; (**E**,**F**)—distribution data from 1961 until 2021 and forecasts of bioclimatic variables from the climatic model CNRM-ESM2-1 2021 (**G**,**H**)—distribution data from 1961 until 2021 and forecasts of bioclimatic variables from the climatic model MIROC6; (**A**,**C**,**E**,**G**)—for training and test (25%) data; (**B**,**D**,**F**,**H**)—for 20 replicates with cross-validation.

**Figure 7:**
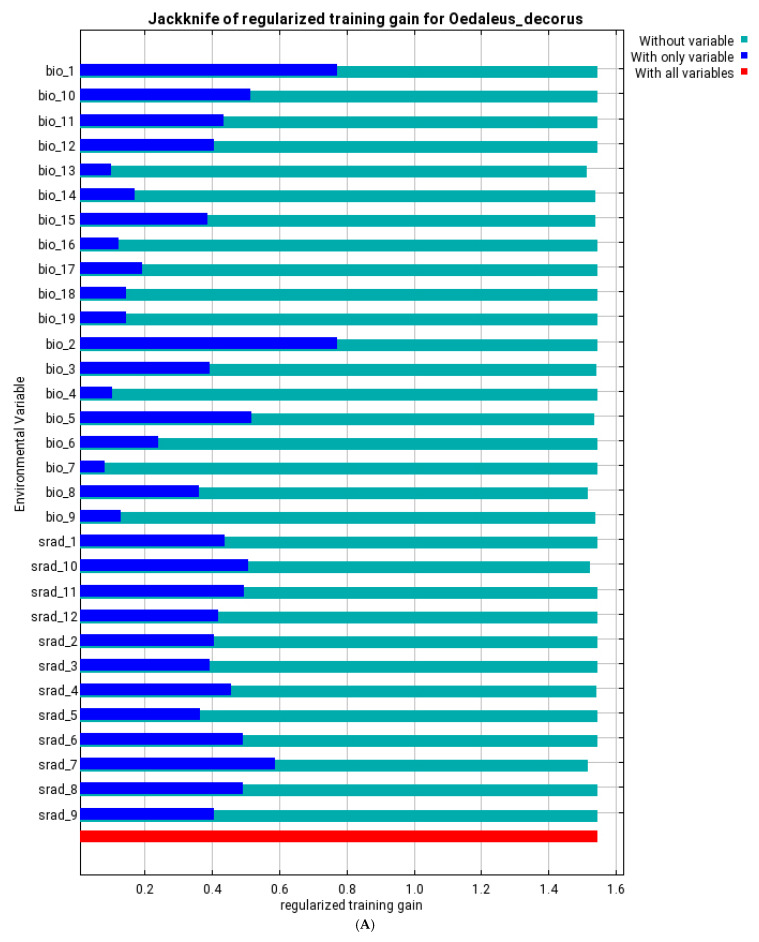

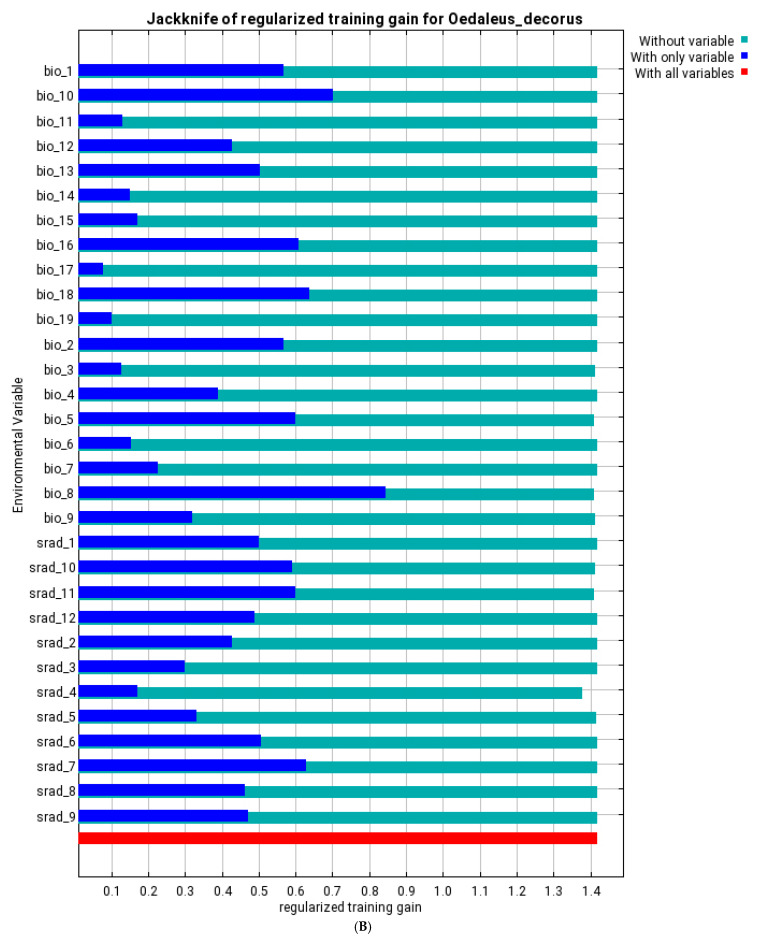

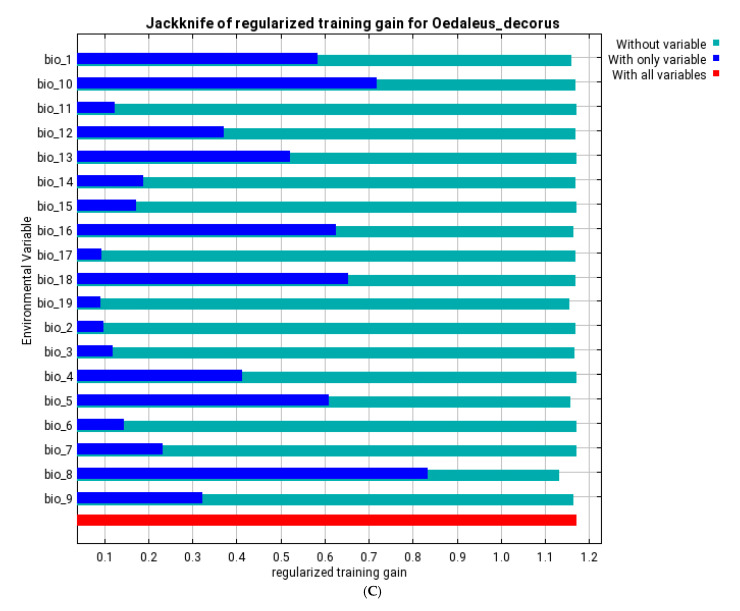

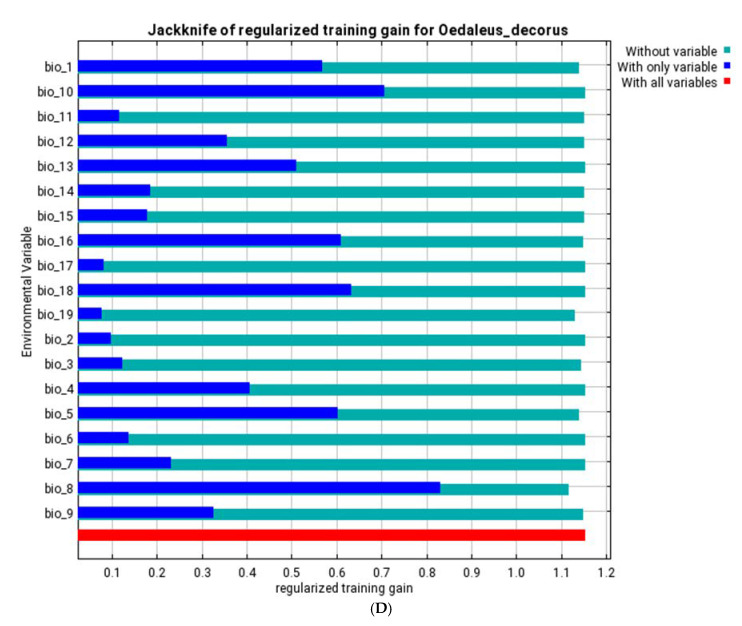
Jackknife of regularized training gain for the *Oedaleus decorus* distribution models for the south-eastern part of the West Siberian Plain: (**A**)—distribution data before 1961 and bioclimatic variables for 1970–2000; (**B**)—distribution data from 1961 until 2021 and bioclimatic variables for 1970–2000; (**C**)—distribution data from 1961 until 2021 and forecasts of bioclimatic variables from the climatic model CNRM-ESM2-1; (**D**)—distribution data from 1961 until 2021 and forecasts of bioclimatic variables from the climatic model MIROC6.

The model performance is high (Figure 6A,B), because the AUC value for training data is 0.954, for the test set—0.871, and for 20 replicates—0.901. In this model, the solar radiation in July is the most important factor (Table 1), besides, the solar radiation in October and April, the annual mean temperature and the precipitation of wettest month are significant as well. The Jackknife test allows to add also the mean diurnal range of temperatures (Figure 7A). Almost all factors are essential for development of nymphae and adults (and certainly for grass vegetation as well), while the solar radiation in October may be associated with autumnal high survival rates of eggs in eggpods.

**Figure 8:**
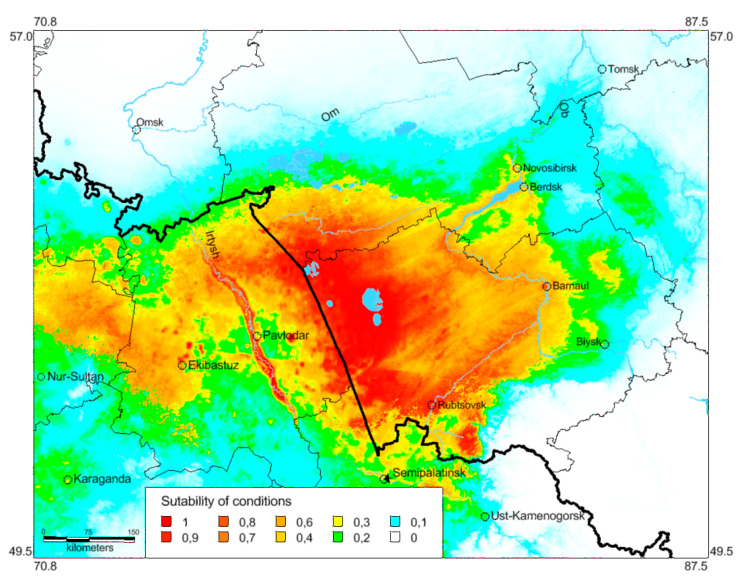
Predicted probabilities of suitable conditions for *Oedaleus decorus* in the south-eastern part of the West Siberian Plain (distribution data from 1961 until 2021 and bioclimatic variables for 1970–2000; point-wise mean for 20 replicates).

The modern known distribution of *O. decorus* (for 1961–2021) matches to the predicted probabilities of suitable conditions (cf. Figure 1 and Figure 8). All steppes and the southern parts of the forest-steppes in Novosibirsk and Altaj Regions of Russia and Pavlodar Region of Kazakhstan are applicable for the handsome cross grasshopper. In the local steppes, high levels of habitat suitability show opportunities of the species upsurges. Besides, there are large areas on the eastern side of the Ob River where populations of this species may occur. Importantly, it can disperse across these territories by dry verges and lawns [64]. Perhaps, *O. decorus* is able to use some vehicles for migrations as well (cf. [59]). 

The performance of this model is also high (Figure 6C,D) (the AUC values for training and test data are 0.935 and 0.922, respectively; for 20 replicates—0.908). For this set of data, two factors, namely, the mean temperature of the wettest quarter and the solar radiation in July, are the most important factors. Three other factors are relatively significant: the solar radiation in October, November, and April. The Jackknife test allows to add the mean temperature of the warmest quarter as well (Figure 7B). One can hypothesize that the first factor and the mean temperature of warmest quarter may determine grass development and may be indirectly associated with grasshoppers’ sustainability. Autumnal and vernal solar radiation can be important for high survival rates of dormant eggs. Importantly, this group of factors looks more significant than prior to 1960. Such a shift may be explained by global and local warming, especially in the end of the 20th century, and by a corresponding increase of warm season duration.

**Figure 9:**
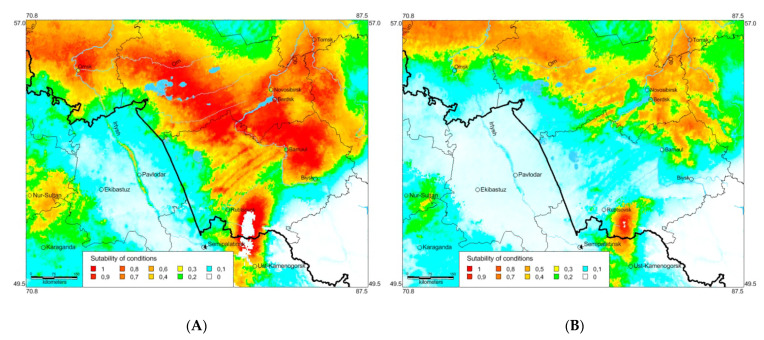
Predicted probabilities of suitable conditions for *Oedaleus decorus* in the south-eastern part of the West Siberian Plain for 2021–2040 (distribution data from 1961 until 2021 and forecasts of bioclimatic variables from the two global climate models CNRM-ESM2-1 [48,49] (**A**), and MIROC6 [50,51] (**B**), and for the 3–7.0 Shared Socioeconomic Pathway based on high greenhouse gas emissions [52]; point-wise mean for 20 replicates).

Ecological modelling of the species distribution in 2021–2040 based on two climatic models (CNRM-ESM2-1 and MIROC6) for the 3–7.0 Shared Socioeconomic Pathway based on high greenhouse gas emissions (Figure 9) shows that the local part of the range may significantly shift northwards and north-eastwards and *O. decorus* will able to distribute over the territories of the modern forest-steppes and south taiga. Besides, it will able to penetrate into two new regions, Tomsk and Kemerovo, as well. Actually, these possible changes may be mainly associated with some significant shifts of life zones northward and north-eastward.

According to the MIROC6 model, the species distribution will shift further north, but the level of condition suitability will decrease. The difference between results of ecomodelling for 2021–2040 may be explained by some peculiarities of the last model, because it can slightly overestimate thermal trends in some regions of Asia [66]. 

Performance of these predictions may be relatively high (the AUC value for training data is 0.910 and for the test sets—0.895; for 20 replicates—0.874 (MIROC6) and 0.888 (CNRM-ESM2-1)) (Figure 6E–G). The main factor for both models is the mean temperature of the wettest quarter, the same as for the contemporary period. Several other factors are also relatively significant: the maximal temperature of warmest month, the mean temperature of warmest quarter, the precipitation of wettest quarter, the annual mean temperature, and the mean temperature of the driest quarter (Table 1). This means, in the near future, the main factors determining the species distribution will remain almost the same. All these factors determine chiefly development of the species during summer. 

In July of 2021, we tried to collect some field data supporting these models. We crossed the central part of the so-called Baraba steppe (actually the forest-steppe region between Omsk and Novosibirsk) from the Kochki settlement in the southern forest-steppe (54.3° N 80.4° E) to the Chulym settlement in the northern one (55.13° N 81.03° E) (Table S2). We found no populations of *O. decorus* along this transect. 

### 3.4. Peculiarities of the Long-Term Dynamics of Species Populations

In the 1920s, the abundance of *O. decorus* was relatively low. Bey-Bienko [41] noted that this species was rather numerous only on the sandy dunes in the southern part of the Kulunda steppe in Kazakhstan, and was rare in some adjacent habitats with dominance of short grasses and sagebrushes. However, *O. decorus* was very common in the semi-deserts of East Kazakhstan. 

In the end of the 20th century and in the beginning of the 21th century, the handsome cross grasshopper became very abundant in the steppe habitats, especially during warm and dry summers. During the Italian locust outbreaks, its abundance could be very high and comparable with the abundance of *Calliptamus italicus* (Table 2). The Spearman rank-order correlation coefficient for the long-term dynamics of populations of *O. decorus* and *C. italicus* is relatively high for each plot studied (Table 2) and very high if we summarize data for all plots (r_s_ = 0.917, *p* < 0.001). 

We also estimated correlations between average densities of both species, but for relatively short rows of observations after acridid outbreak (2004–2008, 2015, and 2018) (Table 3). In this case, the Spearman rank-order correlation coefficient is non-significant for each plot and is moderate, but significant for summarized data (r_s_ = 0.446, *p* = 0.049). 

This means that the contemporary long-term dynamics of the *O. decorus* local populations resembles the dynamics of the Italian locust. As a result, these two species may be members of one temporal distribution guild [67]. 

### 3.5. Frequency of the Color Morphs during the Species Outbreak

**Figure 10:**
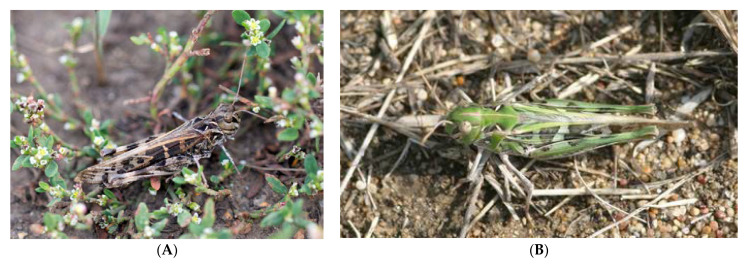
Main color forms of *Oedaleus decorus* in the Kulunda steppe: (**A**)—brown; (**B**)—green.

The handsome cross grasshopper is characterized by high variability in size and color pattern; however, two main color forms, namely brown and green, are very common (Figure 10). Cease et al. [54] tried to check possible associations between color form frequencies, population density levels, and migratory polyphenism for the Mongolian locust (*O. decorus asiaticus*). We analyzed our data (unfortunately, only several limited series) to estimate characters of correlation between frequencies of the brown and green forms and the species abundance in field conditions.

**Figure 11:**
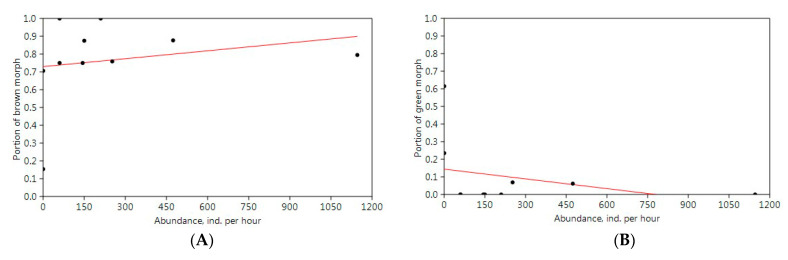
Multivariate regression model for two main color form variations of *Oedaleus decorus* in the Kulunda steppe: (**A**)—brown; (**B**)—green.

The multivariate regression model shows very week non-significant correlations between these parameters: however, the correlation is positive for the brown form (r = 0.214, *p* = 0.553) and negative for the green one (r = –0.325, *p* = 0.359) (overall statistics: R^2^ = 0.070, MSE = 0.050, Wilks’ lambda = 0.853) (Figure 11).

Our estimation conforms with the observations for *O. d. asiaticus* [54]. The brown morph is more or less common despite level of abundance. During species outbreaks, a population may consist only of brown adults; however, when the species abundance is very low, the portion of this morph can be very high as well (Figure 11A). The green individuals present in populations with low or moderate abundances (Figure 11B) and in low proportions. However, in other regions, there are several common color forms, e.g., the grey one [68]. Besides, the species demonstrates typical cryptic behavior [68]. This means that diversity of its color patterns may be determined by specificity of a habitat, and individuals of *O. decorus* can select microlandscapes depends on their color forms [68], though Vorontsovsky [9] emphasized that, in the Orenburg Region, the color forms of *O. decorus* were not associated with specific types of habitats. 

## 4. Discussion

Species distribution model approaches have been used to discuss and solve some issues related to species conservation problems and invasions. Several models were generated for grasshoppers and other orthopteran groups (e.g., [69,70,71]); however, there are a few publications concerning possible pest acridids. Olfert et al. [72] tried to model the distribution shifts of the economically important species, namely the migratory grasshopper or *Melanoplus sanguinipes* (F.), in North America. Recently, two articles concerning ecomodelling of the famous desert locust (*Schistocerca gregaria* (Forsk.) were published [73,74]. 

*O. decorus* is a good species for ecomodelling. It is very widely distributed in the southern part of the Palaearctic Region, from south and central Europe, through north Kazakhstan, south Siberia and Mongolia to north and north-east China, and from North Africa, through Asia Minor, Levant, Caucasus, Iran, Kopet Dagh (Turkmen-Khorasan Mountain Range), north Afghanistan, Tien Shan, Pamiro-Alay, south Kazakhstan, Xinjiang, to E China [1,3,4,5,6,7,8]. The species is also found on the Canary Islands and on Madeira [3]. In Europe, the northern boundary of its range crossed France, Switzerland, Slovakia [3], north Ukraine, and European Russia (Orel, Tambov, and Samara Province) [75]. Uvarov [75] also noted that the species occurred in the Moscow Province; however, this mention is uncertain [76]. In the steppes of south Siberia, the species was distributed over their western (up to Kurgan and Omsk) (*O. d. decorus*) and eastern parts (Minussinsk and Transbaikal areas—*O. d. asiaticus*) [76]. 

In the end of the last century and in the beginning of the 21th century, the species distribution over Central Europe did not change significantly [77]. *O. decorus* was also found in the Lipetsk and Tambov Regions of European Russia [78]. The northern boundary of its range remained almost the same in the mountains of South Siberia as well [1,4,5,59]. However, in the Volga River Basin (the eastern part of European Russia), in the second half of the 20th century, several sporadic colonies of this species were found in the taiga life zone of the Kirov Region [79]. In 2014, the species was also found in the Kologrivski Forest Nature Reserve (the taiga life zone, Kostroma Region, about 58°N) [80]. *O. decorus* also became widely distributed in the Republic of Tatarstan [81]. Similar changes are described for the south-eastern part of West Siberian Plain as well (see Results). Such changes, especially the relatively wide dispersal of the species through territories on the eastern side of the Ob River, were predicted more than 30 years ago [4,64].

*O. decorus* is the usual and often abundant form in dry grasslands of extra-tropical Eurasia, including human-dominated landscapes. It is well represented in many collections, and their known localities are relatively numerous. In this context, importantly, our studies are restricted territorially, and we used several relatively limited sets of data (see Section 2). This is why our results should be interpreted as the preliminary ones. 

*O. decorus* has become one of the most common and widely distributed species not only in the dry steppes of the south-eastern parts of West Siberian Plain, but in the typical and meadow steppes of the region as well. During upsurges the density of its adults is commonly about one to two per square meter. However, our data show that sometimes its density may be more than ten adults per square meter. In many cases, some overlapping of outbreaks of the Italian locust and *O. decorus* was observed, especially in the dry steppes and the semi-deserts of Russia and Kazakhstan. As a result, these two species may damage almost all spectrum of cultivated plants, pastures, and hayfields. Further, in the steppes, during outbreaks hoppers and especially adults of *O. decorus* can actively migrate from one habitat to others as well [21,22]. 

The main trends in the species distribution over the region are supported by ecological modelling. Our results show that the main ecological factors determining the species distribution are the factors defining its development in the middle of summer. Importantly, our analysis demonstrates that the monthly averaged levels of solar radiation can be very useful variables for forecasts. It is understandable, because really the level of solar radiation can be characterized as a complex factor, which influences on both species development and net primary production. Continuation of global warming may result in further northward and north-eastward shifts of range boundaries of *O. decorus* and in changes of distribution and abundance of its populations, especially in the optimal parts of its population system where the species occupies normally all suitable habitats, its average abundance is relatively high and its colonies are common even during depressions [31,82]. In this context, it is important that *O. decorus asiaticus* has become one of most important pests in the eastern parts of the steppe life zone [22,23,24,54,83,84], and a similar possible scenario may be realized for *O. decorus decorus* in the south-eastern part of the West Siberian Plain in the nearest future. 

## 5. Conclusions

Since the 1960s, in the south-eastern parts of the West Siberian Plain, the distribution pattern of the handsome cross grasshopper changed significantly. Nowadays, *O. decorus* occupied almost all local steppes and the southern part of the forest-steppes, up to 55°N, and spread over the forest-steppes on the eastern side of the Ob River too. These changes may be explained by both climatic changes (especially northward) and some transformations of human activities (mainly for eastward spreading). One can suppose that in the nearest future, this species will continue to spread eastward and penetrate into the so-called Kuznetsk steppe in the Kemerovo Region. Furthermore, the ecologo-geographic modelling demonstrates evident opportunities for *O. decorus* to penetrate northwards (at least up to 58°N) and north-eastwards as well.

The species has become very common and occurs in almost all steppe habitats in the steppe life zone and on plots with xerophytic and mesoxerophytic vegetation (especially overgrazed meadows, roadsides, and dry lawns) on the eastern side of the Ob River. In 1999–2002, the abundance of *O. decorus* was very high in the Kulunda steppe between the Irtysh and Ob Rivers. This period of its long-term dynamics may qualified as the local outbreak. It is vitally important that local populations of *O. decorus* often became very abundant in the same habitats and in the same periods as the populations of the Italian locust—one of the most important pests in the region. In this case, the situation for plant protection services may be very complicated, because the handsome cross grasshopper (*O. decorus*) is more or less a typical grass feeder, while the Italian locust prefers dicots. This means during joint outbreaks, these two species can simultaneously damage almost all plants. Besides, one can also suppose that local steppe colonies of *O. decorus* may evolve and some gregarization processes may develop, resulting in their transformation to more or less typical locust populations as described for populations of the Mongolian locust (*O. decorus asiaticus*) in Inner Mongolia [23,54,83,84]. 

## Figures and Tables

**Table 1:** Predictive contributions for two periods (until 1960 and 1961–2021) and for two climatic models for 2021–2040.

Variable	Variable Explanation	Distribution Until 1960		Distribution 1961–2021		CNRM-ESM2-1		MIROC6	
		Percent Contribution	Permutation Importance	Percent Contribution	Permutation Importance	Percent Contribution	Permutation Importance	Percent Contribution	Permutation Importance
bio_1	annual mean temperature	**10.9**	12.4	0.4	1.3	**5.1**	17.1	3.2	18.4
bio_2	mean diurnal range (mean of monthly (max temp—min temp))	2.7	25.7	0.1	0.9	0.3	0.5	0.2	1.2
bio_3	isothermality (bio2/bio7) (×100)	3.5	1.5	1.8	1.1	0.2	0.1	0.6	0.2
bio_4	temperature seasonality (standard deviation ×100)	0.1	0	0.2	0	0.1	0	0.1	0.1
bio_5	max temperature of warmest month	1.4	2.1	5.5	4.5	**7.6**	11	**9.9**	13.9
bio_6	min temperature of coldest month	0	1.1	0	0	0	0	0	0
bio_7	temperature annual range (bio5-bio6)	0.2	1.4	0.1	1	0.1	0.1	0.1	0.1
bio_8	mean temperature of wettest quarter	1	0.3	**31.3**	17.9	**57.9**	26.9	**60.4**	21.3
bio_9	mean temperature of driest quarter	0.7	2.4	0.4	0.3	3.8	0.1	**3.5**	0.4
bio_10	mean temperature of warmest quarter	0.3	0	0.2	0.4	**6.4**	1.2	**6.2**	0.3
bio_11	mean temperature of coldest quarter	0	0	0	0	0	0.2	0	0.1
bio_12	annual precipitation	0.4	0.1	0	0.1	0.2	4.8	0.2	1.9
bio_13	precipitation of wettest month	**8.7**	8.4	2.4	0	2.5	0.1	2.1	0.3
bio_14	precipitation of driest month	2.4	1.8	0.1	0.5	0.5	2.4	0.5	3.1
bio_15	precipitation seasonality (coefficient of variation)	0.8	0.6	0.6	0.8	0.2	1.8	0.4	3.1
bio_16	precipitation of wettest quarter	0.1	0	2.2	5.9	3.6	19.2	3.3	18.9
bio_17	precipitation of driest quarter	4.1	0.1	0	0.1	1.4	0.9	1.7	0.9
bio_18	precipitation of warmest quarter	0	0	2.4	1.4	**5.9**	0.9	**4.3**	1.1
bio_19	precipitation of coldest quarter	0.5	0.1	0.8	0.3	4.1	12.6	3.3	14.8
srad_1	solar radiation, January	0	0	0.1	0.4	n.a.	n.a.	n.a.	n.a.
srad_2	solar radiation, February	0	0	0	0	n.a.	n.a.	n.a.	n.a.
srad_3	solar radiation, March	0	0	0	0	n.a.	n.a.	n.a.	n.a.
srad_4	solar radiation, April	**11.9**	0.6	**4.7**	25.3	n.a.	n.a.	n.a.	n.a.
srad_5	solar radiation, May	0	0	0.1	1.7	n.a.	n.a.	n.a.	n.a.
srad_6	solar radiation, June	0.3	0	0.3	0	n.a.	n.a.	n.a.	n.a.
srad_7	solar radiation, July	**36.9**	30.4	**25**	0.4	n.a.	n.a.	n.a.	n.a.
srad_8	solar radiation, August	0	0	0.8	0	n.a.	n.a.	n.a.	n.a.
srad_9	solar radiation, September	0		0	0	n.a.	n.a.	n.a.	n.a.
srad_10	solar radiation, October	**12.8**	11.1	**13.4**	22.9	n.a.	n.a.	n.a.	n.a.
srad_11	solar radiation, November	0.4	0	**7.1**	12.7	n.a.	n.a.	n.a.	n.a.
srad_12	solar radiation, December	0	0	0	0	n.a.	n.a.	n.a.	n.a.

In bold and highlighted green—five most significant variables for each group; n.a.—not available.

**Table 2:** Dynamics of the abundance (ind. per hour) of *Oedaleus decorus* relative to the abundance of the Italian locust in the Kulunda steppe.

Year	Aleksandrovskij	Yarovoe	Ust-Volchikha
2000	1146/2682	252/582	474/144
2001	210/246	54/450	24/234
2002	6/0	0/24	72/42
2003	6/+	0/4	6/6
2004	0/+	0/4	0/+
2005	0/24	54/6	0/0
2006	0/18	60/30	6/0
2007	18/84	150/60	20/+
2008	60/78	144/6	40/0
2015	+/+	4/0	204/480
2018	+/6	?	17.2/17.1
Spearman’s correlation	0.586 (*p* = 0.058)	0.609 (*p* = 0.063)	0.518 (*p* = 0.102)

Abundance of *O. decorus*/abundance of the Italian locust; +—one or several specimens were found beyond counts (in such cases, the value 0.01 was used to compute correlations); ?—no data; years of acridid outbreak (commonly associated with the Italian locust) are in bold (cf. [32,33]).

**Table 3:** Dynamics of the average density (ind. per m^2^ ± s.e.) of *Oedaleus decorus* relative to the average density of the Italian locust in the Kulunda steppe.

Year	Aleksandrovskij	Yarovoe	Ust-Volchikha
2004	0/0.32 ± 0.32	0.32 ± 0.16/+	0/0.32 ± 0.32
2005	0.43 ± 0.30/0.32 ± 0.23	0.13 ± 0.13/0.38 ± 0.22	0/+
2006	0/+	0.53 ± 0.24/0.21 ± 0.15	0.16 ± 0.16/0.16 ± 0.16
2007	0.48 ± 0.32/0.32 ± 0.23	0.96 ± 0.48/0.64 ± 0.32	0.16 ± 0.16/+
2008	0.42 ± 1.06/0.48 ± 0.27	0.27 ± 0.18/0.51 ± 0.25	0.32 ± 0.32/ 0
2015	+/0	0/0	0.64 ± 0.64/ 1.28 ± 0.89
2018	0/+	?	0.32 ± 0.32/0.32 ± 0.32

Density of *O. decorus*/density of the Italian locust; +—one or several specimens were found beyond counts (in such cases, the value 0.0001 was used to compute correlations); ?—no data.

## Data Availability

Not applicable.

## Supplementary Materials

The following are available online at https://www.mdpi.com/article/10.3390/insects13010049/s1, Table S1. Studied localities for all acridid species and known records of *Oedaleus decorus* before 1960. Table S2: Studied localities for all acridid species and known records of *Oedaleus decorus* after 1960.
